# Difficulties faced by healthcare workers in PPE use during covid-19 pandemic: A mixed-method study from a tertiary hospital in India

**DOI:** 10.6026/973206300214338

**Published:** 2025-12-15

**Authors:** Aishwarya Vijay Kamble, Sakeyt Changdeo Khobragade, Vasudha Belgaumkar

**Affiliations:** 1 Department of Respiratory Medicine, House Officer Respiratory Medicine King Edward Memorial Hospital Mumbai, India; 2 Department of Orthopaedic, King Edward Memorial Hospital Mumbai, India; 3 Department of Dermatology, B.J. Government Medical College, Pune, Maharashtra, India

**Keywords:** Protocol breach, professional burnout, dermatological issues, donning and doffing, ergonomic design, pandemic preparedness, occupational health and safety, awareness, Healthcare workers, covid-19 outbreak, personal protective equipment (PPE)

## Abstract

A mixed-method cross-sectional study at Sassoon General Hospital, Pune, assessed PPE-related challenges among 257 HCWs between August and December 2021. Nearly all HCWs (99.2%) reported discomfort, primarily excessive sweating (95.3%), headaches (60.3%), communication barriers (56.8%) and feelings of suffocation (52.1%). Over half experienced improper sizing and dermatological issues, while 32.7% struggled with donning and doffing despite prior training. Almost half (48.7%) admitted breaking PPE protocol due to discomfort and 31.5% tested positive for COVID-19 while on duty. Thematic analysis highlighted ergonomic inadequacies, prolonged wear, harsh environmental conditions and psychological burden, underscoring the urgent need for climate-appropriate, user-friendly PPE and strong institutional support.

## Background:

The COVID-19 pandemic brought unprecedented challenges to healthcare systems worldwide, exposing healthcare workers (HCWs) to high infection risks and emotional stress. According to the World Health Organization (WHO), the use of personal protective equipment (PPE) was central to infection prevention and control (IPC) strategies in all healthcare settings during the pandemic [1]. PPE protects HCWs from direct exposure to infectious agents, especially respiratory droplets and aerosols that transmit SARS-CoV-2 [2-3]. Despite its protective role, prolonged PPE use has led to substantial physical, emotional and operational strain on HCWs. Documented issues include heat stress, dehydration, pressure-related injuries, communication difficulties and fatigue [4, 5- 6]. These effects are exacerbated in resource-limited and tropical settings, such as India, where environmental conditions amplify discomfort and operational inefficiencies [7].

A Cochrane rapid review also highlighted barriers to PPE adherence, such as design-related challenges, lack of training and insufficient breaks [8]. Historically, the evolution of PPE has been shaped by prior pandemics and occupational hazards, ranging from plague masks to modern respirators and gowns [9, 10]. Current PPE includes gloves, N95 masks or equivalents, goggles, face shields, gowns and shoe covers, which must meet national and WHO-defined standards to ensure efficacy and safety [1, 11]. Yet, many healthcare institutions lack user-centered PPE designs or sustainable reuse protocols [12]. Given the limited research combining physical, psychological and experiential aspects of PPE burden in Indian settings, our study aimed to comprehensively evaluate challenges faced by HCWs. Conducted at Sassoon General Hospital, Pune, this mixed-method study seeks to identify difficulties and propose frontline-informed improvements in PPE design, training and institutional policies.

## Aims and objectives:

[1] To identify the difficulties encountered by health care workers while using PPE to determine any factors contributing to various difficulties

[2] To understand health care worker's perspective regarding the use of PPE

## Materials and Methods:

This cross-sectional, mixed-method study was conducted at B.J. Government Medical College and Sassoon General Hospital, Pune, a designated tertiary care COVID-19 center in Maharashtra, India. The study period spanned from August to December 2021. Prior approval was obtained from the Institutional Ethics Committee (Ref: IEC/2021/124). Participation was voluntary and written informed consent was taken from all individuals. Confidentiality was maintained throughout and no identifiable personal data were recorded. The study population included healthcare workers (HCWs)-comprising senior and junior doctors, nurses, interns and ancillary staff-who were directly involved in the care of COVID-19 patients. To be eligible, participants had to be posted in COVID-19 wards or intensive care units (ICU) and must have used personal protective equipment (PPE) for at least two weeks at a stretch. HCWs who declined consent or had incomplete forms were excluded from the analysis. A semi- structured offline questionnaire, as depicted in Table 1 (see PDF), was developed to capture demographic data, pre-existing co- morbidities, patterns of PPE use (eg. Duration and frequency of duty shifts), difficulties encountered and health issues experienced (including dermatological, respiratory, or systemic symptoms). The questionnaire was validated for content and construct validity by two independent experts. Internal consistency was ensured through pilot testing. Multiple-choice responses were used to standardize answers and minimize inter-observer bias. Data collection was conducted in-person during hospital shifts, ensuring minimal disruption of duty. A subset of participants was purposively sampled for in-depth personal interviews to gather qualitative insights on operational difficulties, emotional burden and adaptation strategies. Interviews were conducted in a confidential setting and recorded anonymously with verbal consent. Data from completed questionnaires were entered manually into Microsoft Excel and later imported into IBM SPSS Statistics for Windows, Version 26.0 (Armonk, NY: IBM Corp.) for analysis. Interview transcripts were manually coded and reviewed independently by two investigators to ensure consistency in qualitative theme extraction.

## Statistical analysis:

Descriptive statistics were used to summarize demographic and outcome variables. Continuous variables were expressed as mean ± standard deviation (SD) or median with interquartile range (IQR), depending on data distribution assessed via the Shapiro-Wilk test. Group differences were assessed using independent t- test or Mann-Whitney U test for continuous variables and Chi-square or Fisher's exact test for categorical variables. Correlation between continuous variables was assessed using Pearson or Spearman correlation coefficients. A two-tailed p-value <0.05 was considered statistically significant. Thematic analysis was performed for qualitative data to identify patterns related to PPE-related challenges, operational issues and emotional responses.

## Results:

Out of 320 healthcare workers approached, 270 completed the offline questionnaire. After data cleaning and de-duplication, 257 responses were included in the final analysis. The mean age of participants was 34.5 years (range: 20-60), with 63.76% aged between 21 and 40 years. A majority were female (63.4%). Most respondents were staff nurses (57.6%), followed by resident doctors (30.7%), interns (10.1%) and others including assistant professors and technical staff. PPE usage was nearly universal. Face masks (99.2%), gloves (97.7%), gowns (95.7%) and head cover (92.6%) were the most commonly used components. Despite high compliance, only 36.89% reported a satisfactory fit and quality of N95 masks. Additionally, 52.9% indicated that the PPE provided was not of appropriate size. A significant number (51.8%) reported wearing PPE for over 6 hours daily, with 65.8% performing duty stretches of 1 week. Cumulative PPE use of more than 6 months was reported by 41.73% of respondents. Training in donning and doffing was received by 81.7% of participants; however, 32.7% reported difficulties. Common issues included confusion about the correct sequence, ill-fitting PPE and spatial constraints in designated areas. Common comorbidities included skin and respiratory allergies (7.4%), asthma (5.1%), hypertension (3.9%) and diabetes (2.3%). Only 4.6% had pre-existing dermatological diseases. As depicted In Table 2 (see PDF), nearly all respondents (99.2%) experienced discomfort, while working in PPE. Figure 1 (see PDF) demonstrated the types of issues such as, Excessive sweating (95.3%), headaches (60.3%), communication difficulties (56.8%) and suffocation (52.1%) were prominent. Other issues included itching (41.2%), thirst (54.5%), breathlessness (36.6%) and neurological symptoms like tingling and lack of concentration.

Dermatological complaints included skin irritation (55.6%), acne (31.1%), contact dermatitis (14%) and dyspigmentation (5.8%). Logistic difficulties included inability to access washrooms (73.5%), falls (45.9%) and near-falls due to shoe covers (44.4%). Despite this, only 25.2% used any preventive measures. Notably, 48.7% admitted to breaking PPE protocol due to physical distress and 31.5% had tested COVID- positive while on duty. Seasonal patterns showed that summer was the most problematic season (92.6%), followed by monsoon and winter (both 16.3%). Sweating, breathlessness and skin issues were notably worse during summer. Cross-tabulations revealed that most symptom distributions (e.g., sweating, breathlessness, dermatological issues) were not significantly associated with age, gender, designation, or duration of duty (p > 0.05). However, presence of underlying disease showed significant associations with headache (p < 0.0001), dermatological issues (p < 0.0001) and itching (p = 0.002).

## Qualitative findings:

Thematic analysis of interviews revealed concerns clustered around poor PPE design, physical and psychological strain and inadequate operational support. Specific themes included:

[1] Non-breathable materials and fogging of eyewear.

[2] Inadequate fit and discomfort during prolonged use.

[3] Emotional fatigue, burnout and communication barriers with patients.

[4] Concerns regarding reused or damaged PPE.

Despite these challenges, many HCWs emphasized the importance of PPE and expressed resilience and commitment to patient care.

## Discussion:

The COVID-19 pandemic imposed an extraordinary burden on healthcare systems worldwide, with frontline healthcare workers (HCWs) facing prolonged exposure, high workloads and elevated infection risk. Although the peak of the pandemic may have passed, the emergence of new cases-reportedly around 23,000 per month globally at the time of writing this manuscript-underscores that COVID-19 remains a persistent public health threat. Moreover, the world continues to witness outbreaks of both emerging and re-emerging infectious diseases, including Zika virus, Ebola, avian influenza and various occult bioterrorism risks. Since 1970, nearly 40 such infectious threats have been identified, highlighting the omnipresent risk of future pandemics. Thus, the need for high-quality, user-friendly and situation-adapted PPE remains an essential component of healthcare preparedness.

Personal protective equipment (PPE), while indispensable in preventing transmission, has emerged as a significant source of physical and psychological strain. Our study highlights the high prevalence of PPE- related discomfort and its association with operational lapses, potentially compromising infection control [1, 2]. While existing literature has documented the difficulties associated with PPE use, most studies rely on online surveys and retrospective data collection, which may fail to capture the depth of frontline experiences. This study aimed to address that gap by conducting face-to-face interviews and direct observations of HCWs in a high-stress clinical environment. By engaging directly with participants, the study was able to elicit rich, contextual insights into the real-world impact of PPE on workflow, physical comfort, communication and mental well-being. The professional distribution ensured a representative understanding of PPE-related issues across healthcare tiers. The results detailed PPE usage patterns. The majority of participants did not report major comorbidities, indicating a generally healthy cohort. While allergies were the most common, pre-existing dermatological diseases were rare, with only 11 out of 241 respondents. This suggests that most PPE-related dermatological complaints reported later in the data were largely new onset or aggravations. In our cohort, nearly all HCWs reported discomfort with PPE. Excessive sweating (95.3%), headaches (60.3%) and suffocation (52.1%) were common, reflecting findings from Santoro *et al.* [13], who documented widespread pressure injuries, heat stress and dermatological issues globally. These findings are reinforced by a review from Phan *et al.* which reported a significant frequency of contact dermatitis, acne and skin breakdown due to prolonged mask use [14]. Extended duty hours and seasonal heat compounded the problem. In summer, breathlessness and dermatological complaints were most prevalent, as also reported in a study from CDC during hot-humid months [6]. Although 81.7% of our participants were trained in donning and doffing, nearly one-third faced difficulties, the most frequently cited issue was a lack of clarity regarding the correct sequence of various components, in line with observations by Westermann *et al.* [15], who identified time constraints, poor layout of PPE stations and discomfort as barriers to proper protocol adherence. This discrepancy suggests that formal instruction alone may be insufficient in ensuring procedural safety. Issues such as confusion over the correct sequence, forgetfulness and practical limitations like ill-fitting PPE components or tight shoe covers were frequently noted. These challenges were further magnified by concerns over poor PPE quality specifically material fragility as well as inadequate sanitation resources and suboptimal donning/doffing environments.

The persistence of these difficulties, even among trained and experienced personnel, highlights the need for continued, hands-on and scenario-based training. It also underscores the importance of ergonomic PPE design and the development of better infrastructure, such as spacious and clearly designated donning/doffing stations equipped with mirrors, sanitizers and visual guides. Without such systemic improvements, the risk of self-contamination and compromised infection control remains high, these findings reinforce that PPE effectiveness is more about appropriate use and implementation than just as availability. Interview themes further highlighted mental fatigue, anxiety and moral injury-mirroring global findings from WHO-affiliated burnout reports. While institutions emphasized PPE supply, few addressed ergonomic design or mental health. The reported symptoms spanned multiple organ systems, illustrating that PPE-related stress is not limited to superficial irritation but manifests as a multisystemic burden. Dermatological symptoms, such as sweating, irritation, itching, acne and pressure marks, were widespread and often severe enough to interfere with daily functioning. Respiratory complaints, including suffocation, thirst and breathlessness, further compounded discomfort, especially in hot and humid conditions. Neurological symptoms headaches, difficulty concentrating and sensory disturbances indicate cognitive fatigue and may have implications for clinical decision-making and patient safety. Moreover, the frequent inability to use washrooms due to PPE- related constraints introduces an additional layer of physical strain, with potential long-term implications for musculoskeletal and urological health. Equally concerning are the behavioural consequences Protocol breaches (48.7%) and infection (31.5%) among participants raise alarm. Inadequate PPE sizing, communication barriers and fogging of eyewear led to unsafe practices, echoing findings from multiple cross-sectional surveys [8, 9]. nearly one-third had tested positive for COVID-19 while on duty. These findings directly link PPE-related discomfort to safety lapses and increased risk of infection for the HCWs themselves as well as their patients and colleagues. Despite the severity and prevalence of symptoms, only a minority employed preventive strategies, indicating either a lack of access or awareness, or perhaps a sense of futility given the limited options available. Seasonal variation was a noteworthy modifier, with the summer months being the most difficult-further strengthening the argument for climate-responsive PPE design. Interview themes further highlighted mental fatigue, anxiety and moral injury-mirroring global findings from WHO-affiliated burnout reports. While institutions emphasized PPE supply, few addressed ergonomic design or mental health. The themes identified reflect perceived shortcomings in PPE design, implementation and support infrastructure. In contrast to online studies, our qualitative approach revealed nuanced, lived experiences- connecting physical discomfort with emotional fatigue, communication challenges and protocol breaches. The proactive suggestions by HCWs-ranging from breathable materials to rest breaks-highlight their readiness to engage in meaningful reform. These insights should inform future PPE strategies through participatory design, ergonomic innovation and institutional responsiveness to safeguard frontline health workers and ensure preparedness for future health crises. Low preventive measure uptake (25.2%) also parallels a German survey showing poor use of barrier creams and hydration schedules [16]. Concerns regarding skin diseases in vulnerable individuals (e.g., those with pre-existing eczema or asthma) have been validated in other reviews [17]. A generally healthy, predominantly young cohort experienced such high levels of physical and psychological strain, further reinforcing the need for urgent reform. Discomfort was not merely a side effect-it was a potential vector for compromised care, protocol violation and occupational hazard. These findings argue strongly for the implementation of ergonomic, breathable and user-centred PPE design, along with policies that mandate rest breaks, rotation schedules and access to support resources.

## Strengths and implications:

Our mixed-method offline design ensured contextual depth and real-time clarification-an advantage over online-only surveys. We advocate for design improvements (breathable, fog-resistant, hypoallergenic materials), hydration policies, scheduled PPE breaks and simulation-based refresher training [13, 14, 15, 16-17]. Studies from tropical nations emphasize these as feasible reforms.

## Limitations:

As a single-center, cross-sectional study, generalizability is limited. Subjective reporting introduces potential recall bias. Qualitative saturation was not formally assessed but approached.

## Conclusion:

This study highlights the significant physical, psychological and operational challenges faced by healthcare workers during the use of personal protective equipment (PPE) in the COVID-19 pandemic. Nearly universal discomfort, prolonged exposure times and procedural difficulties underscore the urgent need for improvements in PPE design, usability and institutional support mechanisms. Our findings demonstrate that issues such as ill-fitting PPE, communication barriers and inability to access rest facilities not only impair HCW comfort but also increase the risk of protocol breaches and infections. These challenges are particularly concerning in resource-limited and high-volume settings like India. To enhance healthcare worker safety and maintain quality of care, it is imperative to invest in climate- appropriate, ergonomic PPE, conduct hands-on training and implement structured duty rotations and psychological support systems. By translating frontline feedback into practical reforms, healthcare institutions can ensure that PPE protects without compromising well-being or clinical performance, thus improving preparedness for future public health emergencies.

## Additional information:

## Disclosures:

## Human subjects:

Informed consent for treatment and open access publication was obtained or waived by all participants in this study.

## Animal subjects:

All authors have confirmed that this study did not involve animal subjects or tissue.

## Payment/services info:

All authors have declared that no financial support was received from any organization for the submitted work.

## Financial relationships:

All authors have declared that they have no financial relationships at present or within the previous three years with any organizations that might have an interest in the submitted work.

## Other relationships:

All authors have declared that there are no other relationships or activities that could appear to have influenced the submitted work.
